# Mapping of a Major QTL for *Ceratocystis* Wilt Disease in an F1 Population of *Theobroma cacao*

**DOI:** 10.3389/fpls.2018.00155

**Published:** 2018-02-14

**Authors:** Luciel dos Santos Fernandes, Stefan Royaert, Fábio M. Corrêa, Guiliana M. Mustiga, Jean-Philippe Marelli, Ronan X. Corrêa, Juan C. Motamayor

**Affiliations:** ^1^Plant Sciences, Mars Center for Cocoa Science, Itajuípe, Brazil; ^2^Statistics, Universidade Estadual de Santa Cruz, Ilhéus, Brazil; ^3^Mars, Incorporated, Miami, FL, United States

**Keywords:** SNP markers, cacao breeding, disease resistance, candidate genes, marker-assisted selection

## Abstract

Cacao is an important crop, its beans are key raw materials for the chocolate and cosmetic industries. *Ceratocystis* wilt of cacao (CWC) caused by *Ceratocystis cacaofunesta* is a lethal disease for the crop. Therefore, the selection of resistant cacao varieties is one of the viable ways to minimize losses in cacao production. In this paper, we described the identification of a major QTL associated with CWC in an F1 mapping population from a cross between a resistant, “TSH 1188,” and a susceptible genotype, “CCN 51.” A set of 266 trees were genotyped using 3,526 single nucleotide polymorphic markers and then multiple QTL mapping analyses were performed. Two QTLs were identified on chromosomes IV and VI. The major QTL was located at 20 cM from the top position of chromosome VI, accounting for more than 60% of the phenotypic variation. The favorable allele T1, with haplotype GTT, came from the “TSH 1188” parent. It was evident that the haplotype combination T1C2 on chromosome VI was the most significant for resistance, since 93% of resistant trees had this haplotype. The major QTL converged to a genomic region of 739.4 kb that harbored nine candidate genes, including two major classes of resistance genes, which would make them the primary candidates involved in the resistance to CWC. The haplotypes detected are now used to improve the efficiency and precision of the selection of resistant trees in cacao breeding.

## Introduction

Cacao (*Theobroma cacao* L.), an allogamous member of the Malvaceae, is indigenous to the Amazon rainforest in South America (Motamayor et al., 2002). Cultivated worldwide, cacao is one of the most significant cash crops in many regions, its beans are key raw material for the chocolate and cosmetic industries. Global cacao production reached 3.9 million tons (t) of dry beans in the 2015/2016 (ICCO, 2017). Of this, the Americas supplied 657 thousand t (16.6 %), and Ecuador and Brazil are the largest producers with 232 thousand t (5.85%) and 140 thousand t (3.53%), respectively (ICCO, 2017). Brazilian cacao production has changed slightly over the last 15 years, not only because the area harvested has increased marginally (FAOSTAT, 2017), but also because of abiotic and biotic stresses.

Concerning these biotic stresses, the incidence of several diseases has contributed to decreased production and has led to even further imbalance in the supply and demand of cacao beans. Cacao trees are constantly confronted with important pathogens that target vegetative shoots, pods and flower cushions (*Moniliophthora perniciosa*) (Aime and Phillips-Mora, 2005), pods and leaves (*Phytophthora* spp.) (Barreto et al., 2015; Surujdeo-Maharaj et al., 2016), and the vascular system (*Ceratocystis cacaofunesta*) (Engelbrecht and Harrington, 2005). Of these diseases, *Ceratocystis* wilt of cacao (CWC) is one the most important, mainly because it causes plant death. There is no information about the global impact of CWC, but cacao-producing areas in Bahia, Brazil, with susceptible varieties were highly affected (Lopes et al., 2011). The most affected planting areas were those with progenies derived from the cross between the clonal varieties “SCA 6” × “ICS 1” (Ram et al., 2004; Lopes et al., 2011). The progenies from this cross were named as “Theobahia,” which was widely propagated due to their tolerance to witchs's broom disease and high productivity (Brasil, 2009). However, most progenies from this cross were susceptible to CWC disease, which led to a drastic reduction in cacao production in Bahia, Brazil (Lopes et al., 2011).

The impact of CWC in the cacao production may be diminished by applying effective breeding approaches, such as the mapping of quantitative traits loci (QTL) and marker assisted selection (MAS), to improve the selection of resistant varieties. By means of QTL mapping, it is possible to identify marker–trait associations and investigate the genomic location of candidate genes influencing the trait of interest, along with the quantification of the variation explained by the QTL region (Xu et al., 2017). Furthermore, reliable molecular markers will be available to cacao breeding via MAS, in order to improve the efficiency and precision of conventional breeding. For that, the construction of a dense linkage map for *Ceratocystis* wilt resistance is an essential step for efficient breeding and selection of resistant cacao varieties to CWC.

An F1 mapping population (MP01) created from crosses between two different parents that segregate for CWC has been used to identify QTLs associated with several traits, for instance pod color regulation (Motamayor et al., 2013), witches' broom (Silva et al., 2014; Royaert et al., 2016) and black pod resistance (Bahia et al., 2015; Barreto et al., 2015). Despite CWC being a lethal disease, few studies have focused on discovering molecular markers associated with resistance genes. Recently, simple sequence repeat (SSR) markers were developed in the cacao “Jaca” variety from expressed sequence tags (EST) of tissues infected with *C. cacaofunesta* (Santos et al., 2012a). Moreover, two QTL regions with a small effect, were mapped in a F2 mapping population between “SCA 6” and “ICS 1” (Santos et al., 2012b). However, constructing a linkage map and QTL mapping based on SNP markers for the MP01 population have never been carried out. In this study, we present the results of QTL mapping for CWC in MP01 population. We discuss two genomic regions associated with CWC resistance, and report favorable alleles/haplotypes associated with disease resistance, as well as some potential candidate genes harbored in the QTL regions.

## Materials and methods

### Plant material

“TSH 1188” and “CCN 51,” two cacao varieties highly contrasting for several important traits (Royaert et al., 2016) including for resistance to CWC, were used as parental genotypes to create the MP01 population. “TSH 1188” has been described as resistant to CWC, while “CCN 51” as susceptible (Sanches et al., 2008). Four hundred fifty-nine F1 trees were produced and planted in 2000 in a 3 × 3 m grid (Royaert et al., 2016), under field conditions at Mars Center for Cocoa Science (MCCS), Barro Preto, Bahia, Brazil. Of these trees, 266 trees were selected based on a preliminary study that investigated witches' broom disease resistance (Santos et al., 2007), to be evaluated for CWC resistance. For this study, a CWC resistant rootstock “VB 1151” (Sanches et al., 2008) was produced via seedlings and planted in polyethylene plastic bags of 1.8 dm^3^ filled with a substrate containing soil, perlite and cattle manure at a ratio of 1:1:1. Six months after planting, six replicates per tree were grafted (on the VB1151 seedlings) using plagiotropic shoots from MP01 trees. To prevent any attack by beetles (*Xyleborus* sp.), the site of the experimentation in the greenhouse was treated with 90% commercial calcium oxide. The parents and 266 trees were randomly organized in the greenhouse, with six replicates per tree in the same row.

### Fungal inoculation

*C. cacaofunesta* isolate CF-20 has been described as one of the most aggressive in a previous study (Silva et al., 2007). The CF-20 isolate was grown on Petri dishes containing potato-dextrose-agar (PDA medium) with a pH of 3.0, and then incubated at 24°C for 8 days. Spore suspension was prepared by adding sterile distilled water to the fungal culture, and then scraped with a sterile spatula. Fragments of PDA medium were filtered through sterile gauze, and then the spore concentration was counted with a hemocytometer (Boecoinc, Germany). The final spore concentration was 1.0 × 10^5^ colony-forming unit per milliliter (CFU/mL). The grafted plants were 4 months old when inoculated with 30 μL of spore suspension, in a longitudinal cut of approximately 6 mm in width, 7 mm in length and 1 mm in depth. The inoculation point was 100 mm above the grafting point. The cut was covered with moistened cotton and then wrapped with biodegradable grafting tape (Aglis, Japan).

### Phenotype evaluation

Calculation of disease incidence was based on counting the number of dead plants (NDP), and the disease severity on xylem lesion length (XLL). During the experiment, 12 observations were made according to the following sequence: the first five observations were performed every 4 days after inoculation (DAI); the subsequent seven observations were made every 7 days until 52 DAI. The XLL was assessed when the plant died and at the end of the experiment by cutting the branch laterally, and then measured the highest lesion length along the xylem.

### Experimental design and statistical analyses

The experimental design was completely randomized with the number of replications ranging from four to six per MP01 progeny studied. NDP and XLL data were ranked by calculating the best linear unbiased prediction (BLUP), using a generalized linear mixed model fitted by maximum likelihood with a split-plot in time.

### QTL mapping

The linkage map used for the QTL mapping was the one recently published by Royaert et al. (2016). For the initial detection of QTL with main effects, BLUP data for incidence and severity were analyzed by interval mapping (IM) with MapQTL software, version 6.0 (Van Ooijen and Kyazma, 2009). A significant threshold for logarithm of odds (LOD) was determined by analyzing 1000 permutations with *p*-values of 0.05 (Churchill and Doerg, 1994). The calculated threshold of the LOD score was 3.1 for both traits analyzed. The QTL positions were obtained at the chromosome region of interest, where the LOD score reached its maximum value. LOD score support intervals were also calculated using the interval functions inner 1-LOD and outer 2-LOD, with MapChart software, version 2.3 (Voorrips 2002).Then, a Multiple QTL Mapping (MQM) analysis was performed using the SNP markers closest to QTL peaks as cofactors. The SNP markers selected as cofactors were Tcm004s02747866 and Tcm006s13371871, on chromosomes IV and VI, respectively. Graphical representations of chromosomes containing the QTLs with significant effects and LOD score peaks were drawn using MapChart software, version 2.3 (Voorrips, 2002).

### Identification of haplotypes combination

Phased haplotype data were obtained using JoinMap software, version 4.1 (Van Ooijen, 2006) and iXora (Utro et al., 2013). Haplotypes of SNP markers flanking the QTL regions with significant effects were identified using the SNP data of the trees and MP01 parents. Parental haplotypes were labeled T1 and T2 for “TSH 1188,” and C1 and C2 for “CCN 51.” Then, the chi-square test was carried out (*p*-value of 0.05) to confirm the significant haplotype-phenotype association. The frequency for each haplotype combination was also computed.

### Phylogenetic analysis

The phasing of 131 individuals comprised of 20 individuals from each of five mapping populations (for a total of 100). Three of the mapping populations (MP01, CATIE Type1 and CATIE Type 2) used in the phasing of the haplotypes in the *Ceratocystis* QTL regions are described in Motamayor et al. (2013). One mapping population from Papua New Guinea, consisting of two Trinitario parents (“KA 82” × “K 101”) and the fifth cross includes two diversity panel members, “IMC 67” × “ICS 95.” In addition, a set of 31 diversity panel members from distinct *Theobroma cacao* structural groups was run with fastPhase (Scheet and Stephens, 2006). Two sets of markers were phased; the first set, contained the three markers in the *Ceratocystis* wilt QTL region on chromosome VI. The second set of markers included the three markers plus two extra markers further away on the chromosome. The phased haplotypes using only the three markers and five markers showed consistency in the QTL region. For each individual, relatedness was indicated by the use of a subpopulation index. For example, members of a mapping population were assigned the same index, while unrelated individuals were assigned a distinct index. The use of the mapping populations was solely to be better able to infer haplotypes for the diversity panel members. The expectation-maximization (EM) algorithm for computing the maximum likelihoods were controlled by the following options: 20 random starts, 25 iterations, and 200 haplotypes sampled from the posterior distribution from a particular random start. The default allelic two-parameter error model for inferring true genotypes was also used to scan for genotype errors. Selected representative individuals from each haplotype group, including the parents from the MP01 population, were used to make the neighbor-joining (NJ) tree for the three most significant markers associated with *Ceratocystis* wilt. The distance matrix for phylogeny estimation was created with the Maximum Composite Likelihood algorithm in MEGA v.7.0 with 1000 bootstraps (Kumar et al., 2016).

### Identification of candidate genes

Potential candidate genes for CWC resistance, located between the SNP markers Tcm006s13222057 and Tcm006s13961448 on chromosome VI and between Tcm004s02243097 and Tcm004s02747866 on chromosome IV, were identified from the Matina 1-6 v 1.1 cacao genome database (Motamayor et al., 2013). Then, the protein sequences were compared against BlastP database using (blast.ncbi.nlm.nih.gov/Blast.cgi).The protein-conserved domains were classified in the InterproScan 5 database (Jones et al., 2014), and gene ontology (GO) categories were identified by searching the Interpro database (Mitchell et al., 2015).

### Inoculation of the recombinants

Based on Fast Phase and JoinMap software, phasing of the haplotype data analysis identified seven recombinants trees that displayed recombination events between the maternal and paternal haplotypes in the main QTL region on chromosome VI. We added ten other trees containing the favorable haplotype combination described in **Table 3** (T1C1), and the same quantity for the other three haplotypes combinations (T1C2, T2C1, T2C2). Those plants were used as controls. Besides those, we also included 11 trees that possessed the favorable T1 haplotype, but were phenotypically susceptible (BLUP > 0), and 15 trees with the T2 haplotype, which were evaluated as phenotypically resistant (BLUP < 0). In summary, 76 trees, and the MP01 parents, were selected, grafted onto VB 1151 rootstock (with five replicates each), and inoculated to be study through a second evaluation. The BLUP values from the inoculation of these trees are shown in **Figure 7** in the column name as “Second inoculation.” Moreover, we added the BLUP values obtained from the initial phenotypic evaluation performed for mapping the main QTL on chromosome VI, which are shown in the column “First inoculation” in **Figure 7**.

## Results

### Phenotypic distribution

**Figure 1:**
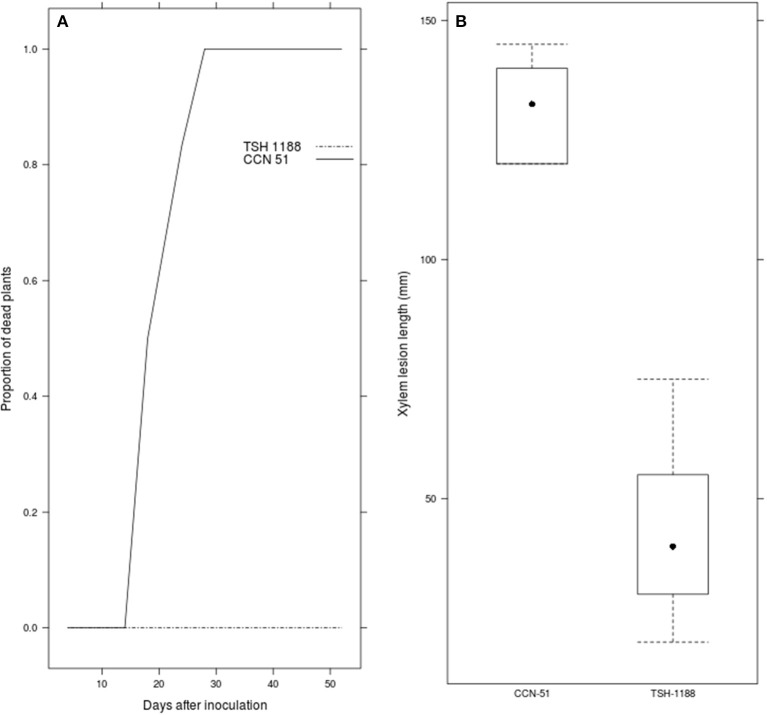
*Ceratocystis* wilt reaction for “TSH 1188” and “CCN 51.” The incidence and severity were assessed over time in the parents for 52 days. The incidence of *Ceratocystis* wilt is presented as the number of dead plants showing disease symptoms for the parents **(A)** The severity in shown as the xylem lesion length **(B)**.

**Figure 2:**
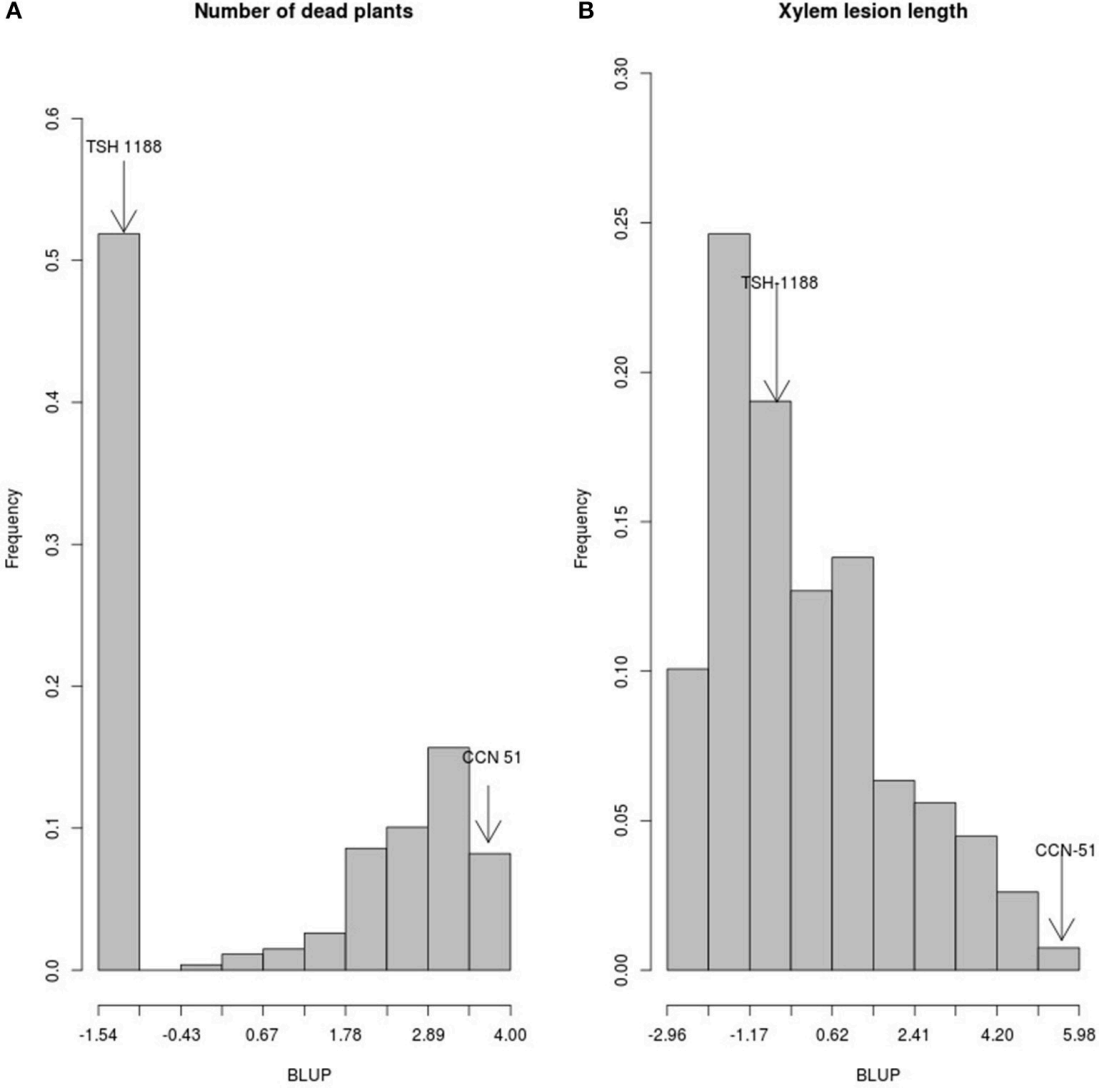
Histogram of frequencies for the numbers of dead plants and xylem lesion length in the F1 population (“TSH 1188” × “CCN 51”). The incidence of *Ceratocystis* wilt is presented as the number of dead plants showing disease symptoms **(A)**. The severity is shown as the xylem lesion length **(B)**. Values of the parents are indicated.

“TSH 1188” showed no symptoms after inoculation, while for “CCN 51” at 18 DAI the NDP was 50%. The number of symptomatic plants increased until 28 DAI (Figure 1A), when NDP was 100%. In contrast, “TSH 1188,” remained symptom-free throughout the whole evaluation period. For “CCN 51,” the XLL mean was higher than “TSH 1188,” 131.67 ± 0.63 mm and 43.33 ± 1.20 mm, respectively (Figure 1B). The individual XLL means in the population ranged from 120.5 to 10.2 mm. The phenotypic values for NDP and XLL indicated that there exists a broad range of resistance or susceptibility (Figures 2A,B). BLUP values less than zero indicated greater resistance. The BLUP values for NDP in the mapping population ranged from −1.51 to 3.98, whereas the BLUP values for “TSH 1188” and “CCN 51” were −1.51 and 3.95, respectively. Two clearly distinguishable groups were identified; one corresponds to the group of 140 completely resistant trees (52.6%), and one group of 126 susceptible trees (47.4%). The susceptible group included 24% of trees with 100% of NDP and 23% that showed NDP ranging from one to three. For XLL, BLUP values showed continuous phenotypic distribution, with values ranging from −2.92 to 5.43 (−0.66 for “TSH 1188” and 5.92 for “CCN 51”). It is noteworthy that for XLL, 159 trees (59.77%) had BLUP values less than zero, of which 124 trees (46.24%) showed BLUP values less than “TSH 1188.” The remaining 107 trees (40.22%) had BLUP values equal to or greater than zero and were classified as susceptible. The *X*^2^ test indicated that the phenotypic segregation ratio fitted a 1:1 ratio for the both traits, suggesting that one or a few genes might control resistance to CWC.

### QTL analysis

**Figure 3:**
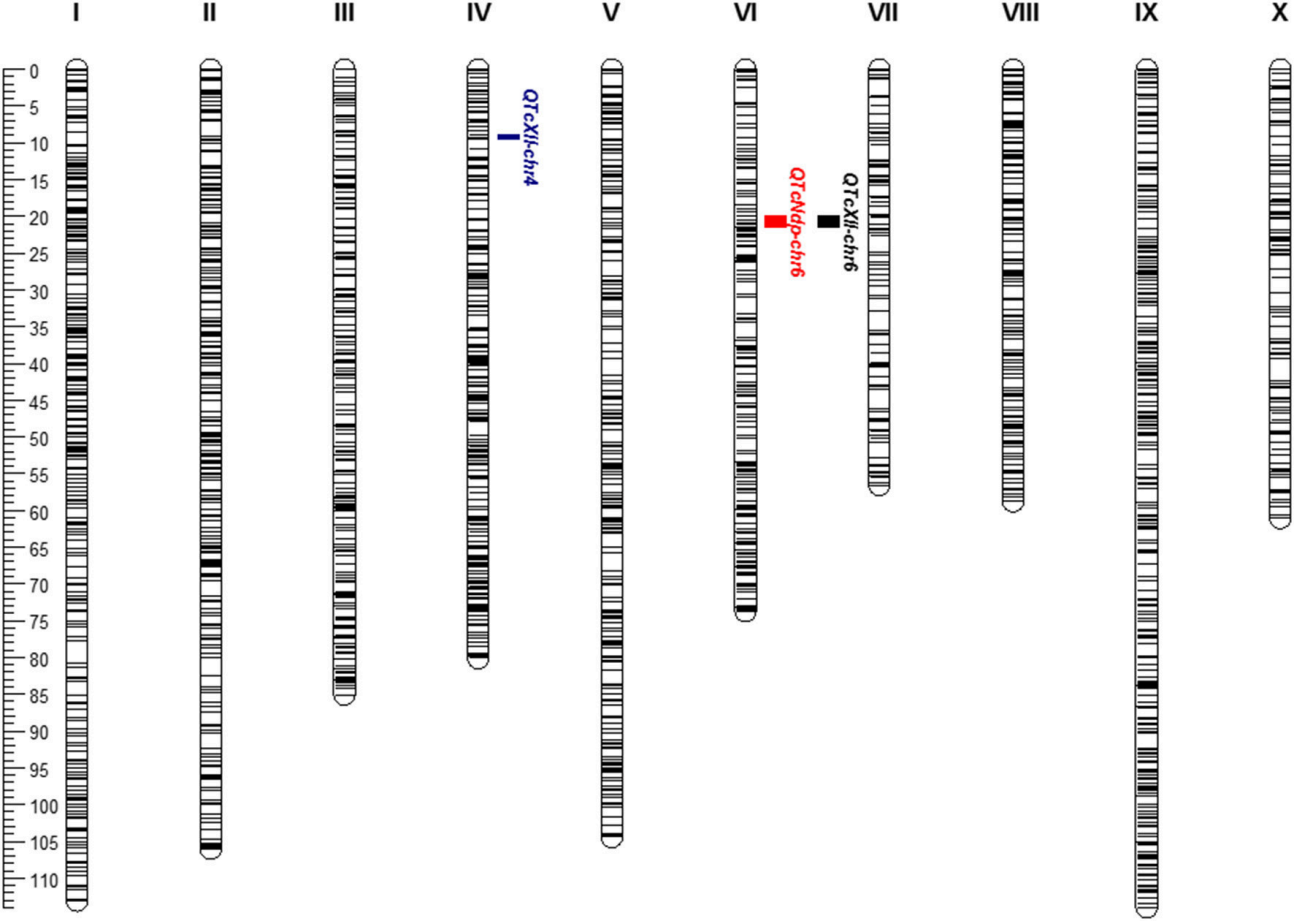
SNP-based linkage map and position of QTLs conferring resistance to CWC in the F1 population (“TSH 1188” vs. “CCN 51”). On the top of the chromosomes are the chromosome number, and the ruler is shown, with the positions of SNP markers in centimorgans (cM). Vertical bars on the right of the chromosome demarcate the location of the QTLs on chromosomes IV and VI.

**Figure 4:**
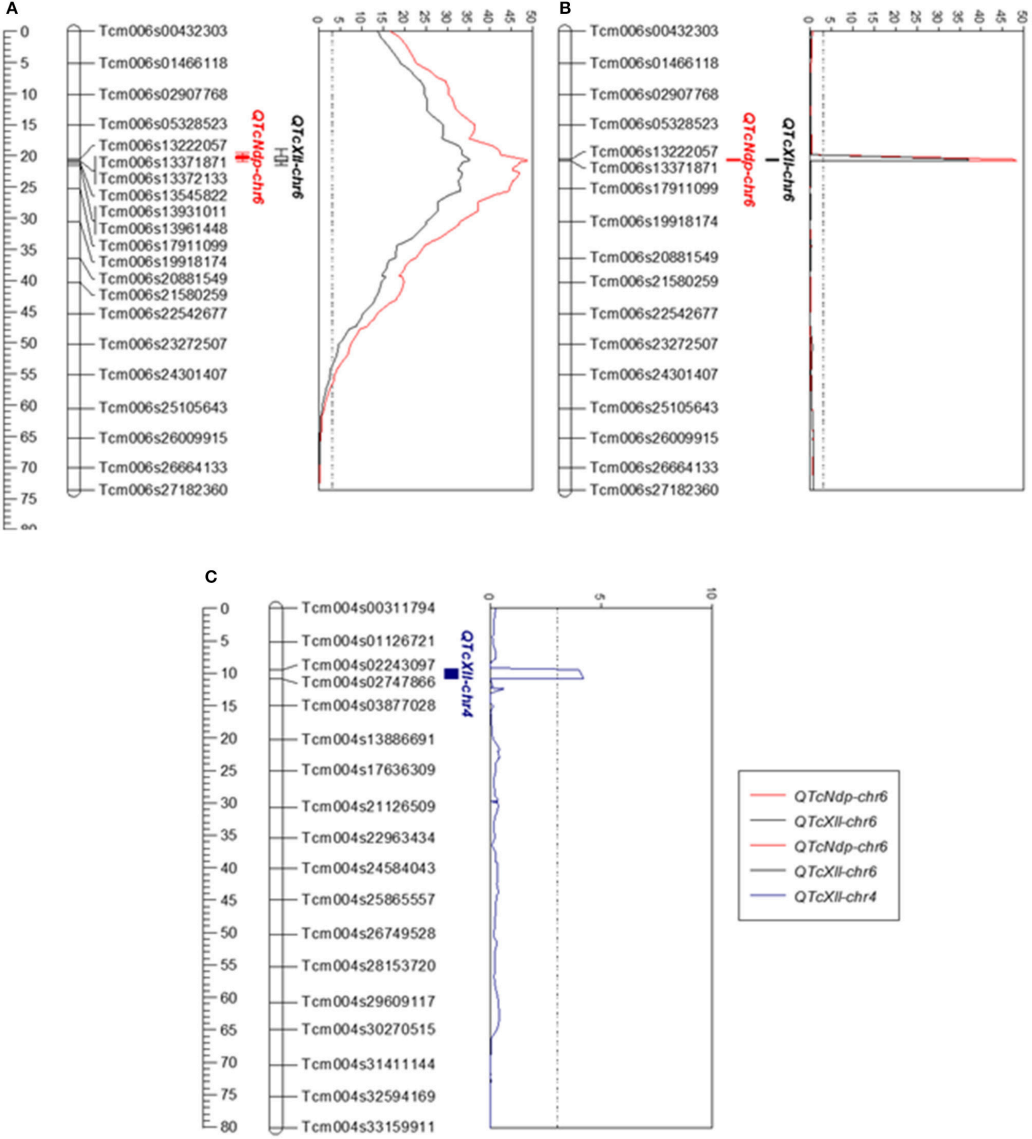
Logarithm of odds (LOD) score peaks for linkage groups VI **(A,B)**, and IV **(C)**, which were significantly associated with resistance to CWC in the F1 population (“TSH 1188” vs. “CCN 51”). The ruler on the left side of the graph shows the position of SNP markers every 5 cM. The graph in **(A)** shows the LOD score plots obtained by interval mapping of NDP and XLL on chromosome VI. The graphs in **(B,C)** show the LOD score plots obtained by multiple QTL mapping of NDP and XLL on chromosomes VI and IV, respectively. The dotted line indicates the LOD significance threshold level of 3.0, calculated by permutation testing (*P* < 0.05). The positions of QTcNdp-chr6, QTcXll-chr6 and QTcXll-chr4 are shown with inner and outer vertical bars for the 1-LOD and 2-LOD support intervals, respectively.

A set of 3,526 SNP markers (Motamayor et al., 2013; Livingstone et al., 2015; Royaert et al., 2016) and 266 trees were used in the linkage mapping construction (Figure 3) and in the QTL mapping analysis. Thirty-seven trees were excluded from the analysis, since genotyping data were not available, resulting in 229 trees used in this step. A major QTL located on chromosome VI was identified by IM and MQM analysis for both traits, and a minor QTL on chromosome IV was identified by MQM analysis for XLL only (Table 1 and Figure 4). The QTL for NDP, closely linked to the SNP markers Tcm006s13371871 and Tcm006s13372133, had a maximum LOD score of 48.02 and explained 62.6% of the phenotypic variation. In the same region, another SNP marker, Tcm006s13222057, was linked to XLL with a LOD score of 35.20, and the phenotypic explanation was 50.2%. MQM analysis for XLL was conducted after the selection of Tcm006s13371811 and Tcm004s02747866 as cofactors, and identified an additional, minor QTL on chromosome IV flanked by Tcm004s02747866 and Tcm004s02243097, which had a maximum LOD score of 4.0 and explained 4.2 and 3.8% of phenotypic variation, respectively.

**Table 1:** Overview of QTLs detected for *Ceratocystis* wilt disease resistance in the F1 mapping population using multiple QTL mapping.

**Chr**.	**QTL name**	**SNP ID**	**cM**	**LOD**	**%**	**“TSH 1188”**		**“CCN 51”**	
						**Allele T1**	**Allele T2**	**Allele C1**	**Allele C2**
IV	*QTcXll-chrIV*	Tcm004s02747866	10.88	4.20	4.00	T	C	T	T
IV	*QTcXll-chrIV*	Tcm004s02243097	9.48	4.00	3.80	T	T	**C**	T
VI	*QTcXll-chrVI*	Tcm006s13222057	20.53	37.07	50.20	**G**	T	T	G
VI	*QTcNdp-chrVI*	Tcm006s13371871	20.81	48.85	62.60	**T**	T	T	C
VI	*QTcNdp-chrVI*	Tcm006s13372133	20.81	48.85	62.60	**T**	C	C	T

*Chr is the abbreviation for chromosome; roman numerals are the identification numbers of the chromosomes; SNP ID is the identification of a single nucleotide polymorphism; LOD is the logarithm of odds calculated for a linkage mapping analysis using the MQM mapping function; the SNP marker position is shown in centimorgans (cM); phenotypic variation explained by the QTL is shown in percentages (%) and SNP alleles for the MP-01 parents, T1 and T2 for “TSH 1188” and C1 and C2 for “CCN 51,” corresponding to alleles 1 and 2, respectively. The haplotypes marked in bold are favorable alleles associated with CWC disease resistance*.

The mapped QTLs were named using a system described for wheat and Arabidopsis (Rant et al., 2013), with modifications. The QTL names consist of the prefix Q followed by the abbreviation of the scientific name, the letter descriptor of the quantitative traits, and the number of the chromosome. Therefore, the QTL on chromosome VI for the NDP was named QTcNdp-chr6, while for XLL was QTcXll-chr6. The minor QTL was named QTcXll-chr4. QTcNdp-chr6 and QTcXll-chr6 corresponded to the same genomic region on chromosome VI.

### SNP haplotype identification and origin of CWC resistance alleles

**Figure 5:**
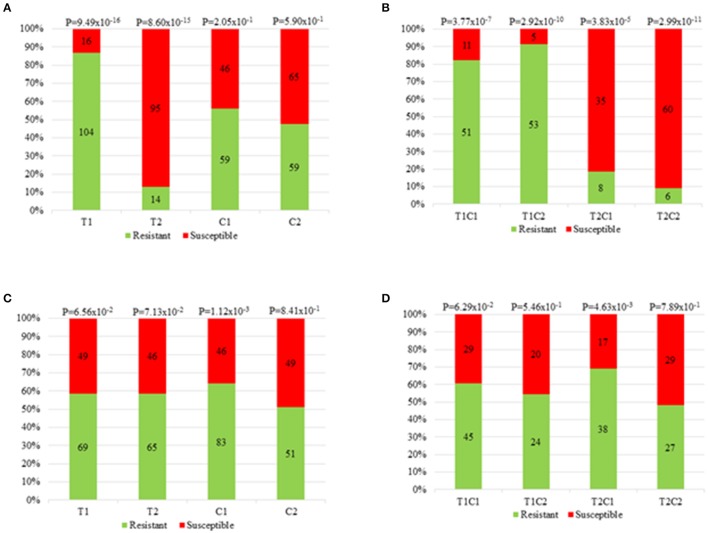
Frequency of resistant and susceptible trees. The frequency of resistant and susceptible trees are given for **(A,C)** each parental haplotype and **(B,D)** are given for each parental haplotype combination for the major QTL on chromosome VI (*QTcNdp-chr6* and *QTcXll-chr6*) and minor QTL on chromosome IV (*QTcXll-chr4*), respectively. The different haplotypes and haplotype combinations are shown on the x-axis, while on the y-axis are shown the percentages of resistant (light green bar) and susceptible trees (light red bar). The *p*-values from the chi-square test for each different haplotype and haplotype combination are shown on the top of stacked bars.

We performed the SNP segregation analysis for the main markers in both QTL regions. The parents' haplotypes were used as reference and the haplotype frequencies of the 266 trees were examined (Table 1). The chi-square test was performed, and the frequency of each allele and haplotype combination was calculated, as shown in Table 2. For QTcNdp-chr6 and QTcXll-chr6, the haplotype T1 (GTT) from the SNP markers Tcm006s13222057, Tcm006s13371871 and Tcm006s13372133 was favorable to resistance against CWC (*P* < 0.05) (Table 1 and Figure 5A). For the combinations T1C1 and T1C2, the frequencies of resistant trees were 82 and 91%, respectively (Figure 5B). However, 16 trees with the GTT resistant haplotype were susceptible, and 13 trees with the TCC susceptible haplotype were evaluated as phenotypically resistant (BLUP < 0) (Table 2).

**Table 2:** Haplotype combinations for the three SNP markers on chromosome VI linked to CWC reactions in the F1 mapping population.

**Hap**.	**SNP ID**			χ2						**% Resistant**
	**Chromosome VI**			**Observed**			**Expected**			
	**Tcm006s13222057**	**Tcm006s13371871**	**Tcm006s13372133**	**Total**	**Resistant**	**Susceptible**	**Resistant**	**Susceptible**	***P*-value**	
T1	**G**	**T**	**T**	120	104	16	60	60	9.49 × 10^−16^	86.67
T2	T	T	C	109	14	95	54.5	54.5	8.60 × 10^−15^	12.84
C1	T	T	C	105	59	46	52.5	52.5	2.05 × 10^−1^	56.19
C2	G	C	T	124	59	65	62	62	5.90 × 10^−1^	47.58
T1C1	**G**	**T**	**T**	62	51	11	31	31	3.77 × 10^−7^	82.26
	T	T	C							
T1C2	**G**	**T**	**T**	58	53	5	29	29	2.92 × 10^−10^	91.38
	G	C	T							
T2C1	T	T	C	43	8	35	21.5	21.5	3.83 × 10^−5^	18.60
	G	C	T							
T2C2	T	T	C	66	6	60	33	60	2.99 × 10^−11^	9.09
	T	T	C							

*Hap is the haplotype of the SNP markers, SNP ID is the identification of the single nucleotide polymorphism marker linked to resistance to Ceratocystis wilt disease on chromosome VI, χ2 is the chi-square test, P-value is the probability value from the chi-square test, and % is the percentage of resistant trees with the respective SNP allele and haplotype. The haplotypes marked in bold are favorable alleles associated with CWC disease resistance*.

The minor QTL, QTcXll-chr4, seemed to have a synergistic effect with the haplotypes T1C1 and T1C2 of the major QTL on chromosome VI (Table 3). The favorable haplotype with an increased number of resistant trees was C1 for the marker Tcm004s02747866, as visualized in Table 3 and Figure 5C. The frequencies of resistant trees were higher when the C1 allele combined with the alleles T1 and T2, forming the haplotype combinations T1C1 and T2C1. The haplotypes T1C1 and T2C1 on QTcXll-chr4 showed frequencies of 60.81% and 69.09% (Figure 5D).

**Table 3:** Haplotype combinations for the two SNP markers on chromosome IV linked to CWC reactions in the F1 mapping population.

**Hap**.	**SNP ID**		χ2						**% Resistant**
	**Chromosome IV**		**Observed**			**Expected**			
	**Tcm004s02243097**	**Tcm004s02747866**	**Total**	**Resistant**	**Susceptible**	**Resistant**	**Susceptible**	***P*-value**	
T1	T	T	118	69	49	59	59	6.56E-02	58.47
T2	C	T	111	65	46	55.5	55.5	7.13E-02	58.56
C1	**T**	**C**	129	83	46	64.5	64.5	1.12E-03	64.34
C2	T	T	100	51	49	50	50	8.41E-01	51.00
T1C1	T	T	74	45	29	37	37	6.29E-02	60.81
	**T**	**C**							
T1C2	T	T	44	24	20	22	22	5.46E-01	54.55
	T	T							
T2C1	C	T	55	38	17	27.5	27.5	4.63E-03	69.09
	**T**	**C**							
T2C2	C	T	56	27	29	28	28	7.89E-01	48.21
	T	T							

*Hap is the haplotype of the SNP markers, SNP ID is the identification of the single nucleotide polymorphism marker linked to resistance to Ceratocystis wilt disease on chromosome IV, χ2 is the chi-square test, P-value is the probability value from the chi-square test, and % is the percentage of resistant trees with the respective SNP allele and haplotype. The haplotypes marked in bold are favorable alleles associated with CWC disease resistance*.

In our study, we identified transgressive trees in the MP01 population for XLL, indicating that there were combinations of favorable alleles from both parents. To support such a statement, we selected 21 trees with BLUP values < 2 for NDP and XLL that did not have the resistant haplotype T1 on chromosome VI. Then, we examined the haplotype combinations on QTcXll-chr4, and performed the chi-square test with 5% of significance. Of these 21 trees, 16 trees had the alleles C/T from “CCN 51” (*p* < 0.016). Therefore, “CCN 51” has a minor but significant effect on resistance to CWC when crossed with “TSH 1188.”

**Figure 6:**
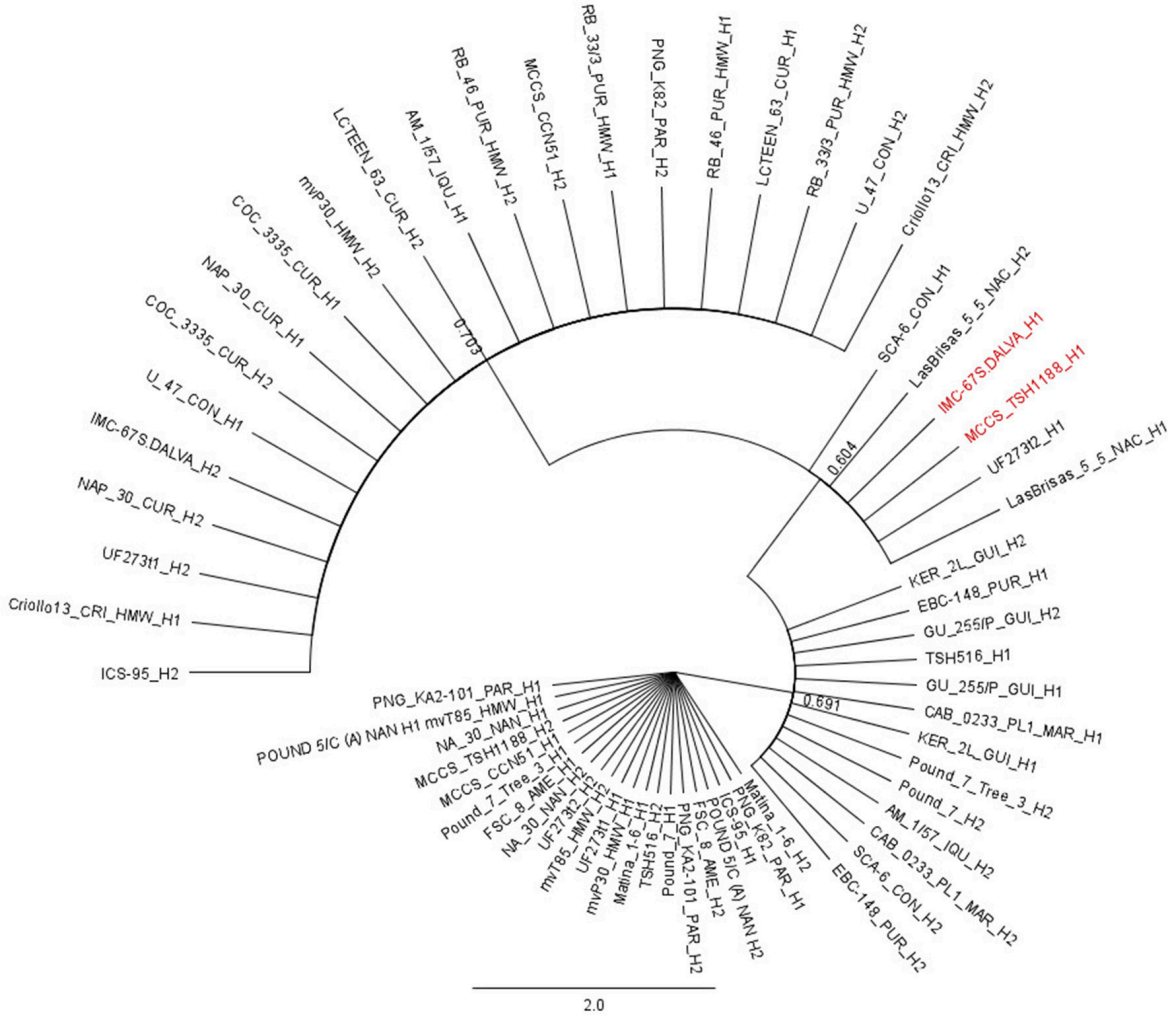
Neighbor Joining tree identifies the origin of the major QTL on chromosome VI (QTcNdp-chr6 and QTcXll-chr6). The red highlighted names are to show that the clones IMC-67.DALVA and TSH 1188 have the same haplotype H1, which was associated with CWC resistance in the MP01.

The resistant haplotype (T1) of “TSH 1188” for the major QTL, which corresponds to the alleles GTT were grouped with the haplotype 1 of the cacao varieties “SCA 6,” “IMC 67,” “UF 273 Type 2” and with both haplotypes of “Las Brisas 5 5” (Figure 6). The second haplotype (T2) grouped with both haplotypes of “Matina 1-6,” “NA 30,” “KA2 101,” “Pound 5/C (A),” and “FSC 8,” “MvT85” and with haplotype C1 of “CCN 51.” The other haplotype of “CCN 51” (C2) grouped with haplotype 2 of “IMC 67” and “ICS 95.”

### Identification of candidate genes

We selected a region on the chromosome VI QTL of 739.4 kbp and a region of 675 kbp on the chromosome IV QTL to study the potential genes regulating CWC resistance. Altogether, we found 338 genes in the two QTL regions, being 159 genes on chromosome IV and 179 genes on chromosome VI. On chromosomes IV and VI, the candidate genes assigned to 12 and 11 GO categories, respectively (data not shown). Biological processes involved in metabolic, cellular and single-organism processes were the most representative for both QTL regions. However, we focused on the stress response category because it involves several important plant resistance reactions, such as defense response, pathogen protein recognition and hypersensitivity responses. In total, we identified 17 genes classified as potentially involved in the category “response to stress” (Table 4). Of these, nine genes were within a 739.4-kbp region in the major QTL QTcNdp-chr6/QTcXll-chr6 and eight within a at 675-kb genomic region in QTcXll-chr4.

**Table 4:** List of candidate genes for *Ceratocystis* wilt resistance identified in the two QTLs on chromosomes VI and IV.

**Gene ID**	**Start**	**Stop**	**Strand**	**aaSize**	**TAIR**	**UniRef**	**Product**	**Interpro classification**
**CHROMOSOME VI**								
Thecc1EG028287t1	13282181	13295487	–	1050	–	Q0E2Y5	Os02g0203500 protein	CC-NBS-ARM
Thecc1EG028297t1	13388379	13392927	–	910	AT5G63020.1	B9GGA8	Cc-nbs-lrr resistance protein	CC-NBS-LRR
Thecc1EG028298t1	13392942	13396745	–	905	AT1G12220.2	B9GGA8	Cc-nbs-lrr resistance protein	CC-NBS-LRR
Thecc1EG028306t1	13471006	13499045	–	1128	AT1G12220.2	B9GGA8	Cc-nbs-lrr resistance protein	CC-NBS-LRR
Thecc1EG028312t1	13539693	13541437	–	261	–	B9GWD4	Uncharacterized protein	Zinc finger, CCCH-type
Thecc1EG028315t1	13553258	13562363	–	145	AT3G15680.1	Q9LW11	At3g15680	Zinc finger
Thecc1EG028293t1	13347157	13372995	–	367	–	B9GWD0	Uncharacterized protein	F-box
Thecc1EG028072t1	10527338	10537904	–	1477	AT1G15520.1	F6HX55	Pleiotropic drug resistance 12.	Plant PDR ABC transporter
Thecc1EG028319t1	13613599	13615422	–	288	AT2G26700.1	Q94E49	Protein kinase PINOID 2	STK
**CHROMOSOME IV**								
Thecc1EG017215t1	2380440	2384563	+	711	AT1G45616.1	B9HZH7	Receptor-like protein 6. sym:AtRLP6	LRR
Thecc1EG017221t1	2388387	2422147	+	1203	AT1G47890.1	B9HZH7	Receptor-like protein 7. sym:AtRLP7 RLP7	LRR
Thecc1EG017172t1	2193702	2197793	–	1025	AT2G34930.1	B9RM78	Serine/threonine-protein kinase bri1	LRR
Thecc1EG017117t1	1943188	1951558	+	764	AT5G14210.1	Q9FMT0	Leucine-rich repeat protein kinase family protein	STK
Thecc1EG017132t1	1995111	1996449	+	170	AT4G08850.1	B9IBE4	Leucine-rich repeat receptor protein kinase family protein	STK
Thecc1EG017134t1	2008502	2016844	+	766	AT3G14470.1	B9ICN9	Cc-nbs-lrr resistance protein	CC-NBS-LRR
Thecc1EG017170t1	2180012	2192435	+	997	AT2G34930.1	B9RM78	Serine/threonine-protein kinase bri1	CC-NBS-LRR
Thecc1EG017277t1	2750829	2755809	+	488	AT5G13160.1	Q9FE20	Serine/threonine-protein kinase PBS1	STK

### Inoculation of the recombinants

**Figure 7:**
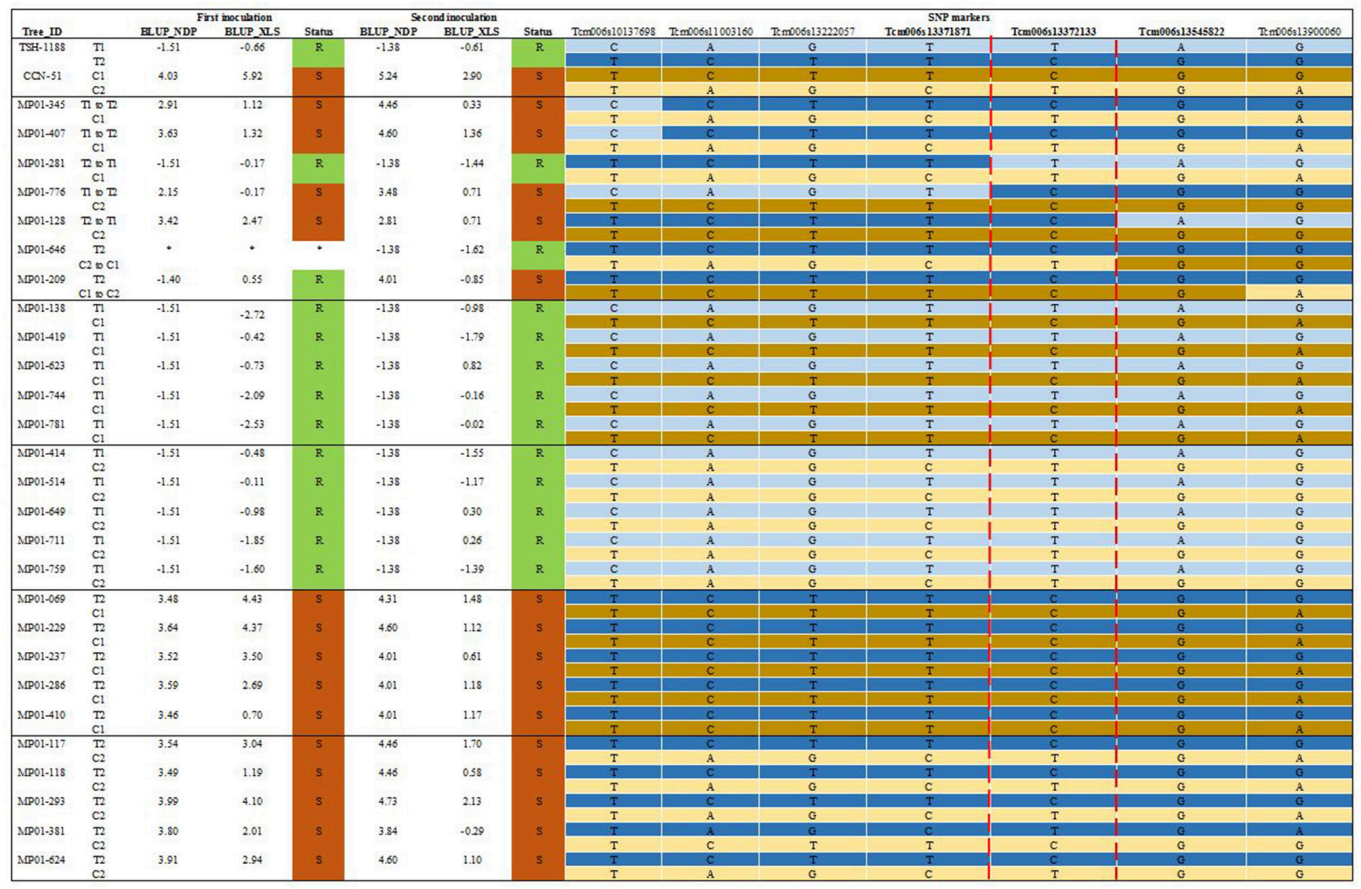
Haplotype combination analysis in the MP01 parents, recombinant trees plus the resistant (R) and susceptible (S) trees. Maternal and paternal haplotypes are shown at the top of the figure. The asterisk (^*^) means that the tree was not evaluated. Hap is the abbreviation of haplotype. The red dash line shows the smallest chromosomic region from T1 associated with resistance. The three main SNP marker are highlighted in bold.

We focused the analysis on seven trees displaying recombination event for the haplotypes in the major QTL region of chromosome VI associated with CWC resistance. In addition, we added flanking four SNP markers, besides the three main markers, to cover an area of 3.762 Mbp and to try to refine the localization of potential candidates genes involved in the CWC resistance by analyzing recombination. The haplotypes of the MP01 parents are shown in Figure 7, highlighting T1 (the haplotype associated with CWC resistance) and T2 for “TSH 1188,” and C1 and C2 for “CCN 51.” In the next block, the seven recombinants are represented. Furthermore, four blocks with representatives of all the different possible haplotype combinations, five per haplotype combination, are shown. In the block with the T1C1 haplotype, all the trees shown were resistant. In the block with the T1C2 haplotype, all the trees shown were resistant as well. In the remaining two blocks with the haplotypes T2C1 and T2C2, all the trees shown were susceptible. These results, as mentioned before, indicate that the haplotype T1 is responsible for the CWC resistance in the MP01.

Of the seven recombinant trees, five trees (MP01-128, 281, 345, 407, 776) possessed a recombination event between the maternal haplotypes in an interval from 10,137,698 to 13,545,822 base pairs (bp). Moreover, within the interval from 13,372,133 to 13,900,060 bp the trees MP01-646 and 209 had a recombination event between the paternal haplotypes, which does not seem to affect the tree resistance.

Three of the five trees with recombination events between the maternal haplotypes helped to narrow down the region even further. The susceptible tree MP01-776 has a recombination event between SNP markers Tcm006s13371871 and Tcm006s13372133, where the haplotype switched from T1 to T2. This indicates that the region upstream of Tcm006s13371871 harbors the genes responsible for the CWC resistance. Another susceptible tree, MP01-128, has a recombination event between SNP markers Tcm006s13372133 and Tcm006s13545822, where the haplotype switched from T2 to T1. This indicates that the region conferring resistance is downstream of marker Tcm006s13545822. Finally, there is the resistant tree MP01-281 that has a recombination event between SNP markers Tcm006s13371871 and Tcm006s13372133, where the haplotype switched from T2 to resistant T1. Together with the trees MP01-776 and MP01-128, this tree MP01-281 confirms that the region conferring resistance is between SNP markers Tcm006s13371871 and Tcm006s13545822.

## Discussion

### Phenotypic distribution for CWC

The phenotypic segregation ratio of 1:1 for NDP (Figure 2A) suggests that only a few genes might control resistance to CWC in the MP01 population. However, the continuous phenotypic distribution of XLL suggests a high level of variability for this disease. BLUP values observed in 150 F1 trees that were lower than in the resistant “TSH 1188” parent (Figure 2B) indicate that genes, likely inherited from both parents, are synergistically acting to increase the resistance level to CWC. A similar observation was reported in a cacao F2 population (“SCA 6” × “ICS 1”) evaluated for CWC (Santos et al., 2012b). The authors observed transgressive segregation for the F2 trees evaluated. Transgressive segregation corresponds to the presence of additive alleles between the parents (deVicente and Tanksley, 1993; Rieseberg et al., 1999). A similar phenotypic pattern in an F1 cacao population was identified for resistance against *Phytophthora palmivora* (Iwaro et al., 1997; Crouzillat et al., 2000), as well as for other crops (Aghnoum and Niks, 2010; Joshi et al., 2013; Tzin et al., 2015).

### QTL and haplotype associated with CWC resistance

MQM analyses identified a major QTL on chromosome VI, QTcNdp-chr6 and QTcXll-chr6, associated with CWC resistance, and a minor QTL, QTcXll-chr4 on IV for XLL. Together, they explained 66% of the phenotypic variation. A previous study identified two QTLs associated with CWC resistance in an F2 cacao mapping population from a cross between “SCA 6” and “ICS 1” (Santos et al., 2012b). These specific QTLs were mapped on chromosomes III and IX, and explained from 6.9 to 8.6% of the phenotypic variation, respectively (Santos et al., 2012b). These values are much lower than what we found for the main QTL on chromosome VI, which accounted for 50.20% for XLL, and 62.60% for NDP (Table 1). On the other hand, the small QTL on chromosome IV explained only 3.80% of the phenotype variation.

The major source of resistance for the QTL mapped by Santos et al. (2012b) might come from “SCA 6,” which is also a well-known source of resistance to witches' broom disease (Bekele, 2004; Maharaj et al., 2011; Royaert et al., 2016). “SCA 6” and “IMC 67” are the great grandparents of “TSH 1188” (Bekele, 2004; Maharaj et al., 2011; Turnbull and Hadley, 2017). “IMC 67” (from the Iquitos genetic group, Motamayor et al., 2008) is a recognized source of resistance to CWC (Turnbull and Hadley, 2017). In our study, the favorable resistant haplotype (T1 = GTT) for CWC was mapped in “TSH 1188” on the major QTL on chromosome VI. This haplotype grouped together with the haplotype 1 of “IMC 67” and some other cacao genotypes (Figure 6). The haplotype combination between T1 (GTT) with C2 (GCT) from “CCN 51” was the most significant to tree resistance against CWC disease in the MP01. This suggests that the alleles inherited from “CCN 51” had synergism with the alleles from “TSH 1188.” Apparently, “CCN 51” has significant additive and dominance effects in inheritance of tolerance to cacao diseases, mainly for witches' broom, in trees having “CCN 51” as a parent (Royaert et al., 2016). These findings might be due to the specific and general combining ability of “CCN 51” (Boza et al., 2012), that contributes for transmitting favorable resistant genes to the trees having “CCN 51” as parental. “CCN 51' is also derived from crosses involving “IMC 67” (Boza et al., 2012), which indicates that “CCN 51” may also have resistance alleles to CWC from IMC67 as TSH1188.

The major effect of the main QTL on chromosome VI was associated with the maternal haplotypes from “TSH 1188.” The results suggested that the trees with the maternal haplotype T1 (GTT) for the SNP markers Tcm006s13222057, Tcm006s13371871 and Tcm006s13372133, are usually resistant to CWC. These data corroborate with the fact that 82 and 91% of the trees possessing the haplotype combinations T1C1 and T1C2 were resistant (Figure 5B and Table 2). However, for some of the trees carrying the T1 haplotype, at least one of the replications died (BLUP > 0). Actually, eleven trees (18%) with the haplotype T1C1, and five trees (9%) with the haplotype T1C2 showed BLUP values higher than zero. Likewise, the situation occurred in which a tree carrying the susceptible haplotype (T2C1 and T2C2) (Table 2) did not show any symptom of susceptibility. In total, 14 trees with haplotype T2C1 and six with T2C2, showed no symptoms of susceptibility. The BLUP values for those trees were less than zero, what classified them as resistant. This reaction can be an escape response from the infection with *C. cacaofunesta*. Even if the inoculum was applied in a uniform and in an optimal concentration (1 x 10^5^ spores/mL), individual trees may have escaped from CWC infection. Moreover, this disease escape reaction could also be associated to scion-rootstock interaction, as mentioned, MP01 progenies were grafted onto seedlings (sexually propagated and genetically different) from a resistant variety used as rootstock.

### Potential candidate genes associated to CWC resistance

We started the identification of candidate genes associated to CWC resistance by covering the whole genomic region of 739.4 kbp, from the markers Tcm006s13222057 to Tcm006s13961448 on chromosome VI. In this region, we identified nine putative candidate genes, encoding proteins with different conserved domains (Table 4). We also covered a genomic region of 675-kbp between the markers Tcm004s02243097 and Tcm004s02747866 on chromosome IV, in which we identified eight candidate genes. Eight genes, three on chromosome VI, see below, and seven on chromosome IV, had homologies with disease-resistance genes encoding for leucine-rich repeat (LRR) proteins involved with specific recognition of pathogen effectors (Zhang et al., 2014; Fenyk et al., 2015), and hypersensitive responses (van Ooijen et al., 2008a,b; Keller et al., 2015). The second major group of genes encoded a serine/threonine protein kinase (STK), which might participate of the signaling cascade (Afzal et al., 2008; Qi et al., 2014), and trigger the disease resistance mechanism in response to pathogen infection (Cao et al., 2011). We also identified a cacao candidate gene, Thecc1EG028287t1, which is a homolog of *Oryza sativa* Os02g0203500, with a CC-NSB-ARM domain involved in programmed cell death (PCD) (Jiao et al., 2012). Other resistant candidate genes include the zinc finger (Gupta et al., 2012) and plant PDR ABC transporter (Nuruzzaman et al., 2014; Sekhwal et al., 2015), as shown in Table 4. The protein containing the F-box domain, Thecc1EG028293t1, might be associated with accumulation of phytohormones in response to pathogen infection (Liu and Xue, 2011; Piisil et al., 2015; Gonzalez et al., 2017; Hedtmann et al., 2017).

Indeed, the phenotype and haplotype analysis of the recombinant trees MP01-776, 182 and 128 in the interval between markers Tcm006s13371871 to Tcm006s13545822 clearly demonstrated that inheritance of the maternal haplotype T1 was highly associated to CWC resistance in the MP01. This recombinant analysis also allowed to narrow down the region associated to the resistance. The two susceptible trees with recombination within the interval between the two markers, MP01-776 and MP01-128, displayed the susceptible haplotype T2. The resistant tree with recombination within the same interval, MP01-281, displayed the resistant haplotype T1 (Figure 7). The haplotype analysis of these three trees reduced the original region on chromosome VI from 739.4 to 174.0 kbp. Within this narrower region, there are five disease resistant candidate genes (among the ones mentioned above), of which three genes (Thecc1EG028297t1, Thecc1EG028298t1, Thecc1EG028306t1) encoded a CC-NBS-LRR resistance protein, one encoded an uncharacterized protein with a Zinc finger-CCCH-type domain (Thecc1EG028312t1), and another one (Thecc1EG028293t1), encoding an uncharacterized protein containing an F-box domain (Table 4). These results also indicated that there might be a copy number variation (CNV) of the candidate genes containing the CC-NBS-LRR domains on chromosome VI. CNV regions are widespread in plant genomes and might have significant associations with phenotypic variations of important traits (Lu et al., 2015), such as disease resistance. This type of genetic polymorphism might play important roles in the expression of disease resistance genes in many crops (McHale et al., 2012; Muñoz-Amatriaín et al., 2013; Lu et al., 2015). The results found in our study may motivate future research aiming to investigate the candidate genes related to CWC resistance.

In summary, the search for QTL regions and molecular markers associated with CWC resistance is one of the major goals of our cacao-breeding program. Accurate QTL mapping relies on an effective and reliable phenotyping data collection, combined with a large set of mapped SNP markers from a cacao segregating mapping population. We have recently published research suggesting that genomic selection may be more powerful than MAS built from QTL mapping approaches for polygenic cacao disease resistance with lower heritability (Navarro et al., 2017). However, in this study we have clearly shown that QTL mapping using SNP markers can be of great benefit when studying cacao disease resistance regulated by fewer genes and with a high heritability. From the SNP data set in the main QTL peak, we were able to identify the favorable alleles/haplotype combinations associated with *Certaocystis* wilt resistance in the MP01 population. Due to an effective phenotyping data collection, and robust SNP data set, we accurately screened a large number of resistant trees carrying a favorable haplotype (GTT). This information, combined with gene annotation data, allowed the identification of potential candidate genes in both QTL peaks. The results from our research are supporting conventional cacao breeding via MAS, in order to select and create new resistant cacao varieties to CWC.

## Author contributions

LF carried out the experimental work, which included experimental design, phenotyping data collection, SNP data analysis and candidate genes identification, produced QTL graphs and wrote the manuscript. SR performed the QTL mapping analysis, assisted with SNP data analysis, and helped editing the manuscript. FC carried out BLUP analysis and produced the graphs. GM carried out the phylogenetic analysis for the SNP markers and created the NJ tree. J-PM and RC was involved in all steps of this research as the advisor of master's student LF. JM provided the SNP markers data, assisted with the SNP data analysis and edited the manuscript.

### Conflict of interest statement

The authors declare that the research was conducted in the absence of any commercial or financial relationships that could be construed as a potential conflict of interest.
